# Endoplasmic stress sensor Ire1 is involved in cytosolic/nuclear protein quality control in *Pichia pastoris* cells independent of *HAC1*

**DOI:** 10.3389/fmicb.2023.1157146

**Published:** 2023-06-20

**Authors:** Yasmin Nabilah Binti Mohd Fauzee, Yuki Yoshida, Yukio Kimata

**Affiliations:** Division of Biological Science, Graduate School of Science and Technology, Nara Institute of Science and Technology, Ikoma, Japan

**Keywords:** unfolded protein response, yeast, *Pichia pastoris*, endoplasmic reticulum, stress response

## Abstract

In eukaryotic species, dysfunction of the endoplasmic reticulum (ER), namely, ER stress, provokes a cytoprotective transcription program called the unfolded protein response (UPR). The UPR is triggered by transmembrane ER-stress sensors, including Ire1, which acts as an endoribonuclease to splice and mature the mRNA encoding the transcription factor Hac1 in many fungal species. Through analyses of the methylotrophic yeast *Pichia pastoris* (syn. *Komagataella phaffii*), we revealed a previously unknown function of Ire1. In *P. pastoris* cells, the *IRE1* knockout mutation (*ire1Δ*) and *HAC1* knockout mutation (*hac1Δ*) caused only partially overlapping gene expression changes. Protein aggregation and the heat shock response (HSR) were induced in *ire1Δ* cells but not in *hac1Δ* cells even under non-stress conditions. Moreover, Ire1 was further activated upon high-temperature culturing and conferred heat stress resistance to *P. pastoris* cells. Our findings cumulatively demonstrate an intriguing case in which the UPR machinery controls cytosolic protein folding status and the HSR, which is known to be activated upon the accumulation of unfolded proteins in the cytosol and/or nuclei.

## Introduction

The endoplasmic reticulum (ER) is an interconnected network of flattened or tubular sacs that are commonly found in eukaryotic cells and serves as a site for the folding and modification of secretory and transmembrane proteins. ER-resident molecular chaperones, such as BiP, assist in the folding of ER client proteins translocated from the cytosol (Pobre et al., 2019). The ER also contains various protein-modification enzymes for disulfide bond formation, such as protein disulfide isomerase (PDI) and Ero1, and for N-linked glycosylation (Braakman and Hebert, 2013). After normal folding and modification, ER client proteins are packed into transport vesicles and transported to the cell surface or other membranous organelles via the Golgi apparatus.

Dysfunction or functional deficiency of the ER is collectively called ER stress, which is frequently accompanied by the accumulation of unfolded proteins in the ER. Excessive load of secretory proteins into the ER is a prominent example of ER stress stimuli. Chemicals or antibiotics that cleave disulfide bonds or inhibit N-linked glycosylation also cause ER stress. In response to ER stress, eukaryotic cells induce a cytoprotective gene induction program called the unfolded protein response (UPR) (Mori, 2009).

The intracellular signaling pathway of the UPR was initially revealed through frontier studies using the yeast *Saccharomyces cerevisiae* as a model organism (Le and Kimata, 2021). Ire1 is an ER-resident type-I transmembrane protein that carries dual enzymatic activities, Ser/Thr protein kinase and endoribonuclease (RNase). Upon ER stress, Ire1 is self-associated and auto-phosphorylated, thus leading to its activation as an RNase that splices the *HAC1* gene transcript (Sidrauski and Walter, 1997; Lee et al., 2008; Ishiwata-Kimata et al., 2013). The spliced form of *HAC1* mRNA is then translated into a transcription factor that induces a number of genes, such as *KAR2* (the BiP-encoding gene), *PDI1* (the PDI-encoding gene), and *ERO1*, many of which support functions of the ER and protein secretory pathway (Cox and Walter, 1996; Travers et al., 2000; Kimata et al., 2006).

In contrast, when not spliced by Ire1, *HAC1* mRNA is poorly translated and virtually functionless at least in *S. cerevisiae* cells (Mori et al., 2000; Rüegsegger et al., 2001; Di Santo et al., 2016). Moreover, according to Niwa et al. (2005), *HAC1* mRNA is the sole target of Ire1. These insights indicate that the functions of *IRE1* and *HAC1* are highly interdependent. As *IRE1* and *HAC1* precisely belong to the same epistatic group, knockout mutations of these two genes exhibit identical phenotypes and do not show additive or synergistic effects (Travers et al., 2000; Schuldiner et al., 2005).

Also in animal and plant cells, Ire1 is involved in the splicing of mRNAs encoding transcription factors, namely XBP1 in metazoans and bZIP60 in plants (Tran and Kimata, 2018). On the other hand, Ire1 promotes the degradation of mRNAs that mainly encode ER client proteins in these species (Coelho and Domingos, 2014). This reaction is called the regulated Ire1-dependent decay (RIDD) and likely contributes to mitigating the protein load to the ER.

Ire1 exclusively performs the RIDD in *Schizosaccharomyces pombe* cells, which do not carry *HAC1*-gene orthologue (Kimmig et al., 2012). In some other fungal species carrying *HAC1*-gene orthologues, Ire1 may also have a role(s) other than splicing of the *HAC1* transcript. According to Feng et al. (2011), the *IRE1* gene (IreA) knockout mutant of the filamentous fungus *Aspergillus fumigatus* exhibits more severe defect in the virulence than the *HAC1* gene (HacA) knockout mutant. Moreover, the *IRE1* knockout mutant of the pathogenic yeast *Candida albicans* was reported to be more sensitive to iron depletion than the *HAC1* knockout mutant (Ramírez-Zavala et al., 2022). Nevertheless, it is unclear how Ire1 functions in these cases.

*Pichia pastoris* (alias *Komagataella phaffi*) has various unique properties that are not seen in *S. cerevisiae* (Ata et al., 2021), while both yeast species belong to the same taxonomic family of *Saccharomycetaceae*. For instance, probably because of the high secretion of endogenous proteins, Ire1 is partially but considerably activated even under healthy growing and non-stressed conditions in *P. pastoris* cells (Fauzee et al., 2020). Also considering that *P. pastoris* is widely used for the production of heterologous secretory proteins (Puxbaum et al., 2015), we believe that the functions of its UPR-related proteins are intriguing research topic. In the present study, we therefore explored the *HAC1*-dependent and *HAC1*-independent physiological roles of Ire1 in *P. pastoris* cells. Our findings cumulatively indicate that in *P. pastoris* cells, Ire1 functions to attenuate cytosolic protein aggregation and the heat shock response (HSR) as well as to perform *HAC1* mRNA splicing.

## Materials and methods

### Genetic manipulation of *Pichia pastoris* cells

We used *P. pastoris* CBS7435 as the wild-type (WT) strain (Küberl et al., 2011). For transformation of *P. pastoris* cells, they were electroporated as described in Wu and Letchworth (2004). Genomic DNA samples were extracted using the Dr. GenTLE kit (Takara Bio, Kusatsu, Japan).

For CRISPR/Cas9-based genome editing, we used the plasmid BB3cK_pGAP_23*_pPFK300_Cas9, which carries the Cas9 nuclease gene, guide RNA expression module, and G418-resistant kanMX marker (Gassler et al., 2019). DNA fragments carrying the guide RNA sequences (Supplementary Table S1) were synthesized by Twist Bioscience (South San Francisco, CA, United States) and ligated with BbsI-digested BB3cK_pGAP_23*_pPFK300_Cas9 using the Gibson assembly kit (New England Biolabs, Ipswich, MA, USA). To generate a donor DNA construct for full-length *IRE1* gene deletion (*ire1Δ0* mutation), 5′- and 3′-flanking regions of the *IRE1* gene were PCR-amplified from genomic DNA using primer sets I1/I4 and I3/I2 (Supplementary Table S2), fused using the Gibson assembly kit, and PCR-amplified again using primer set I5/I6 (Supplementary Table S2). To generate a donor DNA construct for the full-length *HAC1* gene deletion (*hac1Δ0* mutation), 5′- and 3′-flanking regions of the *HAC1* gene were PCR-amplified from genomic DNA using primer sets H1/H6 and H5/H2 (Supplementary Table S2), fused using the Gibson assembly kit, and PCR-amplified again using primer set H7/H8 (Supplementary Table S2). Subsequently, 1 μg of the resulting guide RNA/Cas9 expression plasmid and 5 μg of the resulting donor DNA construct were mixed and used to transform *P. pastoris* cells. The G418-resistant transformant clones were subjected to genomic PCR analysis using primer set I1/I2 or H1/H2 for confirmation of the *ire1Δ0* or *hac1Δ0* mutation and grown in YPD not containing G418 to eliminate the guide RNA/Cas9 expression plasmid.

We also knocked out the *HAC1* gene and *IRE1* gene through genomic insertion of the *kanMX* marker. Using primer sets H9/H10 and H11/H12 (Supplementary Table S3), partial fragments of the *HAC1* gene were PCR-amplified from genomic DNA. The *kanMX* marker was PCR-amplified from BB3cK_pGAP_23*_pPFK300_Cas9 using primer set H13/H14 (Supplementary Table S3). These three PCR products were fused using the Gibson assembly kit and amplified again using the primer set H3/H4 (Supplementary Table S3). Subsequently, the resulting *hac1::kanMX* gene disruption module (*HAC1*-fragment (first half)*-kanMX*-*HAC1* fragment (latter half)) was used to transform *P. pastoris* cells, and the G418-resistant transformant clones were subjected to genomic PCR analysis using the primer set H3/H4 to confirm the *hac1::kanMX* mutation. The zeocin-resistant marker on the *IRE1*-knockout module described in our previous publication (Fauzee et al., 2020) was replaced with the *kanMX* marker to generate the *ire1::kanMX* allele.

The plasmid pAHYB-GFP was previously created for GFP expression from the *AOX1* promoter in *P. pastoris* cells (Yang et al., 2014). The *GAP1* promoter sequence was PCR-amplified from *P. pastoris* genome using oligonucleotide primer sets ccaagcagatctCTCTGCTACTCTGGTCCCAAGTG and ggctacggtaccTGTGTTTTGATAGTTGTTCAATT [capital letters: sequence for annealing to the *GAP1* promoter region, underlined letters: artificially attached restriction sites (BglII and KpnI)]. Then, the PCR product and pAHYB-GFP were digested with BglII and KpnI and ligated, and the resulting plasmid was named pGHYB-GFP, which was used for GFP expression from the *GAP1* promoter. To create the plasmid pGHYB-nGFP, a nuclear localization signal (NLS)-encoding sequence [CCAAAGAAGAAAAGAAAAGTT (corresponding to ProLysLysLysArgLysVal)] was in-frame inserted into the C-terminal position of the GFP-coding region on pGHYB-GFP. After linearization by cutting with BamHI, pGHYB-GFP and pGHYB-nGFP were used to transform *P. pastoris* strains.

### Growth and stress exposure of *Pichia pastoris* cells

For culturing *P. pastoris* cells, we used glucose-based rich medium (YPD medium) containing 1% yeast extract, 2% Bacto peptone, and 2% glucose. Dithiothreitol (DTT) and tunicamycin were purchased from Tokyo Chemical Industry (Tokyo, Japan) and Sigma-Aldrich (Merck KGaA, Darmstadt, Germany), respectively. For agar plates, YPD was solidified with 2% agar. A spectrophotometer SmartSpec 3,000 (BioRad, Hercules, CA, United States) was used to monitor optical density (OD_600_) of the cultures.

Unless otherwise noted, YPD cultures of *P. pastoris* were aerobically shaken at 30°C, and cells in the exponential growth phase were collected. To obtain DTT-treated cells, DTT solution (1 M in water) was added to YPD cultures, which were further shaken at 30°C for 30 min. For the spot growth assay, YPD cultures (OD_600_ = 1.0) were 10-fold serially diluted with YPD, and 1.0 μL of the cell suspensions were spotted onto YPD agar plates.

### RNA analyses

Total RNA samples were extracted from *P. pastoris* cells using the hot phenol method as previously described (Le et al., 2021). For conventional reverse transcription (RT)-PCR analysis to detect *HAC1* mRNA, total RNA samples were subjected to an RT reaction with the *HAC1*-specific RT primer P1, which was followed by PCR with the *HAC1*-specific PCR primer set P3 and P4 in accordance with our previous publication (Supplementary Table S4; Fauzee et al., 2020). Because this PCR traversed the *HAC1* intron sequence, the spliced and unspliced forms of *HAC1* mRNA yielded different-sized PCR products, which were then separated by agarose gel electrophoresis in Tris/borate/EDTA running buffer. Subsequently, ethidium bromide-fluorescent images of the gels were captured using the digital imager E-box (Vilber Lourmat, Marne-la-Vallée, France).

Before RT-quantitative PCR (RT-qPCR) and high-throughput RNA-seq analyses, residual DNA in the total RNA samples was digested with recombinant DNase I (RNase-free; Takara, Kusatsu, Japan) in accordance with the manufacturer’s instruction. Subsequently, DNase I was removed from the total RNA samples by phenol-chloroform extraction and ethanol precipitation.

For the RT-qPCR analysis, total RNA samples were subjected to the RT reaction using poly(dT) oligonucleotide primer (Supplementary Table S4) and PrimeScript II Reverse Transcriptase (Takara, Kusatsu, Japan) as per manufacturer’s instruction. The RT-reaction products were then analyzed by real-time qPCR as described previously (Tran et al., 2019), using the primer sets listed in Supplementary Table S4. The *P. pastoris ACT1* gene transcript was used as the reference (Fauzee et al., 2020), and the ΔΔCt method was used to calculate relative gene expression levels.

High-throughput RNA-seq analysis was performed by GeomeRead Co. Ltd. (Takamatsu, Japan). First, mRNA was purified from total RNA samples using the KAPA mRNA capture kit (KAPA Biosystems, Potters Bar, United Kingdom). Second, libraries were generated using the MGI Easy RNA directional library prep set (MGI Tech, Shenzhen, China) and then analyzed using the DNBSEQ-G400RS DNA sequencer (MGI Tech, Shenzhen, China; 2 × 150 bp paired-end reads, 1 Gb data/sample). Raw FASTAQ data were processed using the CLC Genomics Workbench (Qiagen, Venlo, Netherlands). Reference data for gene mapping and annotation were obtained from the mRNA-seq data have been deposited in DDBJ database under the accession number of PRJDB15162 Pichiagenome.org (http://pichiagenome-ext.boku.ac.at). We used a WEB site-based analyzer, YeastEnrichr (https://maayanlab.cloud/YeastEnrichr/) for the enrichment analysis.

### Protein analyses

After harvesting by centrifugation at 1,600 × g for 1 min, 1.0 = OD_600_ cells were disrupted by agitation with glass beads (425–600 μm) in 100 μL of the lysis buffer containing 50 mM Tris-Cl (pH 7.9), 5 mM EDTA, 1% Triton X-100, and protease inhibitors (2 mM phenylmethylsulfonyl fluoride, 100 μg/mL leupeptin, 100 μg/mL aprotinin, 20 μg/mL pepstatin A, and Calbiochem Protease Inhibitor cocktail Set III (X100 dilution)) and then clarified by flash centrifugation at 750 × g for 30 s. The protein concentration in the crude lysates was determined using the BioRad Protein assay kit (Hercules, CA, USA) and adjusted to 2.5 mg/mL by adding the lysis buffer. Subsequently, the crude lysates were further centrifuged at 8,400 × g for 20 min, and the pellet fractions were washed twice with the lysis buffer supplemented with 2% NP-40.

Protein samples were fractionated by the standard Laemmli SDS-polyacrylamide gel electrophoresis as previously described (Le et al., 2021), and the resulting gels were silver stained using Silver Stain KANTO III (Kanto Chemical, Tokyo, Japan). Alternatively, the gels were subjected to Western blot analysis as previously described (Le et al., 2021). The primary antibodies used were a rabbit anti-ubiquitin antibody (SPA-200) purchased from Stressgen (Enzo Biochem, Farmingdale, NY, United States), a rabbit anti-GFP IgG purchased from MBL Life Science (Tokyo, Japan), and a mouse monoclonal anti-PGK1 antibody 22C5D8 purchased from Abcam (Cambridge, United Kigdom).

### Statistics

Statistical analyses were performed using three independent clones of the same genotype, and values are presented as the means and standard deviations from three biological replicates. To obtain *p* values, we performed a two-tailed unpaired t-test using Microsoft Excel. Alternatively, RNA-seq data were processed using the CLC Genomics Workbench (Qiagen, Venlo, Netherlands). For multiple comparisons of the RT-qPCR data, we performed Dunnett’s two-tailed test, in which probability values (*p* values) less than 0.05 were considered statistically significant.

## Results

### Heat shock response is induced not by the *hac1Δ* mutation but by the *ire1Δ* mutation in *Pichia pastoris* cells

The main research question of this study is whether the *IRE1* and *HAC1* genes have effects on different pathways in *P. pastoris* cells. To this end, we constructed *P. pastoris* cells carrying the *IRE1* knockout mutation (*ire1Δ*) or *HAC1* knockout mutation (*hac1Δ*). Consistent with Gassler et al. (2019), CRISPR/CAS9 technology was used to introduce the *ire1Δ* mutation. The resulting mutant allele carried a full-length deletion of *IRE1* (Supplementary Figure S1A) and is named *ire1Δ0*. Moreover, we initially introduced the *hac1Δ* mutation through insertion of the G418-resistant *KanMX* marker into the genomic *HAC1* gene. The resulting mutant allele carried a full-length deletion of the *HAC1* gene (Supplementary Figure S1B) and is named *hac1::KanMX*.

**Figure 1:**
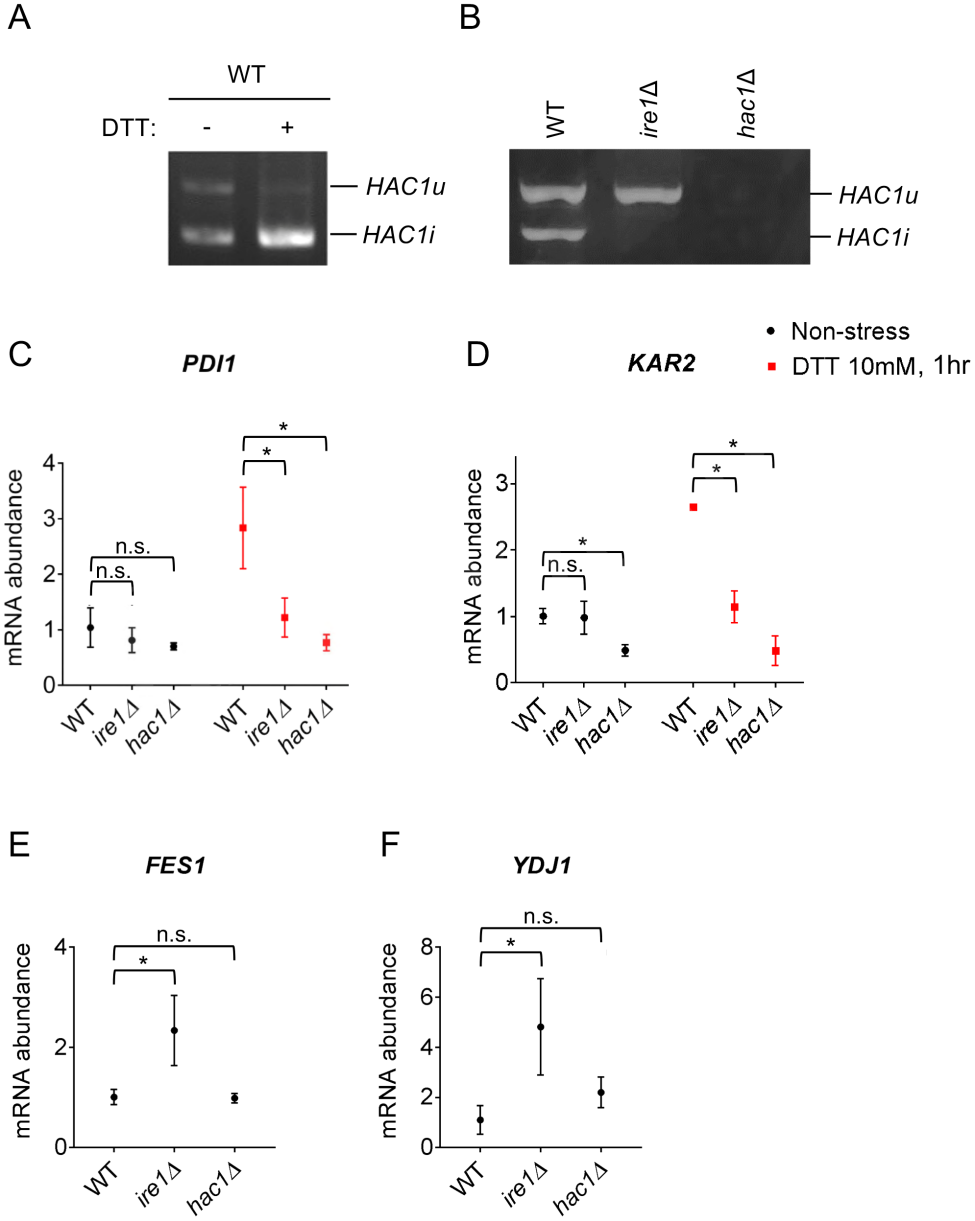
*HAC1* mRNA-splicing and UPR or HSR-marker gene-expression profiles in *P. pastoris* cells carrying the *ire1Δ* mutation or *hac1Δ* mutation. WT, *ire1Δ* (*ire1Δ0*), and *hac1Δ* (*hac1::kanMX*) versions of *P. pastoris* cells were cultured at 30°C under non-stress conditions or stressed with 10 mM DTT for 30 min. **(A,B)** Total RNA samples were subjected to RT-PCR to amplify *HAC1* cDNA variants, which were then fractionated using agarose gel electrophoresis. **(C–F)** Total RNA samples were subjected to RT-qPCR analysis using PCR primer sets that were specific to the indicated genes. Values are presented as relative to that of non-stressed WT cells, which is set at 1.0. Dunnett’s test was performed using the data from WT cells as the control group. n.s.: not significant, *: significantly different (*p* < 0.05).

We previously reported that in WT *P. pastoris* cells, the *HAC1* mRNA is partly spliced even under non-stress conditions (Fauzee et al., 2020). This observation was reproduced in Figures 1A,B. Moreover, *HAC1* mRNA splicing was almost fully induced by the potent ER stressing agent DTT under our experimental conditions (Figure 1A). As described previously (Fauzee et al., 2020) and shown later in this article, not only splicing but also the cellular abundance of *HAC1* mRNA is elevated depending on *IRE1* and ER stress. Although not a quantitative measurement of total *HAC1* mRNA abundance, our data shown in Figure 1A seem to be consistent with this insight. As expected, *HAC1* mRNA was not spliced in *ire1Δ* cells (Figure 1B).

Next, we examined the expression levels of the traditional UPR target genes, *KAR2* and *PDI1*, in *P. pastoris* cells stressed or not stressed by DTT. As previously reported by us and others (Whyteside et al., 2011; Fauzee et al., 2020), DTT induced the expression of *KAR2* and *PDI1* in wild-type (WT) *P. pastoris* cells (Figures 1C,D). Consistent with the widely accepted view that the *IRE1*/*HAC1*-dependent UPR pathway transcriptionally induces the *PDI* gene, the *ire1Δ* and *hac1Δ* mutations almost equally compromised the expression of *PDI1* (Figure 1C). Induction of *KAR2* by DTT was also attenuated by the *ire1Δ* and *hac1Δ* mutations (Figure 1D). However, unexpectedly, only the *hac1Δ* mutation compromised *KAR2* expression under non-stress conditions (Figure 1D).

In *S. cerevisiae* cells, *KAR2* is transcriptionally induced not only by the UPR, but also by the heat shock response (HSR) (Kohno et al., 1993). Therefore, we hypothesized that even under non-stress conditions, the *ire1Δ* mutation, but not the *hac1Δ* mutation, induces the HSR, leading to the higher *KAR2* expression in *ire1Δ* cells than in *hac1Δ* cells. It is widely accepted that the HSR causes the transcriptional induction of genes encoding cytosolic and/or nuclear molecular chaperones and chaperone co-factors (Morano et al., 1998). In this study, we therefore examined the expression of the HSR markers *FES1* and *YDJ1*, both of which encode cytosolic Hsp70 co-factors and are known to be induced by heat shock in other species (Caplan and Douglas, 1991; Kabani et al., 2002; Chen and Qiu, 2020). Intriguingly, the *ire1Δ* mutation, but not the *hac1Δ* mutation, considerably elevated the expression of *FES1* and *YDJ1* under non-stress conditions (Figures 1E,F). In the experiment shown in Supplementary Figure S2, we used another *ire1Δ* allele, namely *ire1::kanMX*, and confirmed that the *ire1Δ* mutation induced *FES1* and *YDJ1*. Similar results as Figures 1E,F were obtained for *HSP42*, *SSA3* (cytosolic/nuclear HSP70 chaperone gene), and *SIS1* (cytosolic/nuclear co-chaperone gene), which are also considered HSR marker genes (Supplementary Figure S3).

### Global gene expression alteration by the *ire1Δ* and *hac1Δ* mutations in *Pichia pastoris* cells

Therefore, the *ire1Δ* and *hac1Δ* mutations likely result in different outcomes in *P. pastoris*. To elucidate this issue more deeply, we performed a transcriptome analysis of *ire1Δ* and/or *hac1Δ* mutant cells. For this analysis, we employed cells carrying CRISPR/CAS9-based gene-deletion mutations (*ire1Δ0* and/or *hac1Δ0*; see Supplementary Figure S1C for the construction of the *hac1Δ0* allele). Heterologous drug-resistance markers were not used because their transcripts could be a bias in the mRNA-seq analysis. To generate cells carrying the *ire1Δhac1Δ* double mutation, cells carrying the *hac1Δ0* mutation were further mutagenized to carry the *ire1Δ0* mutation.

**Figure 2:**
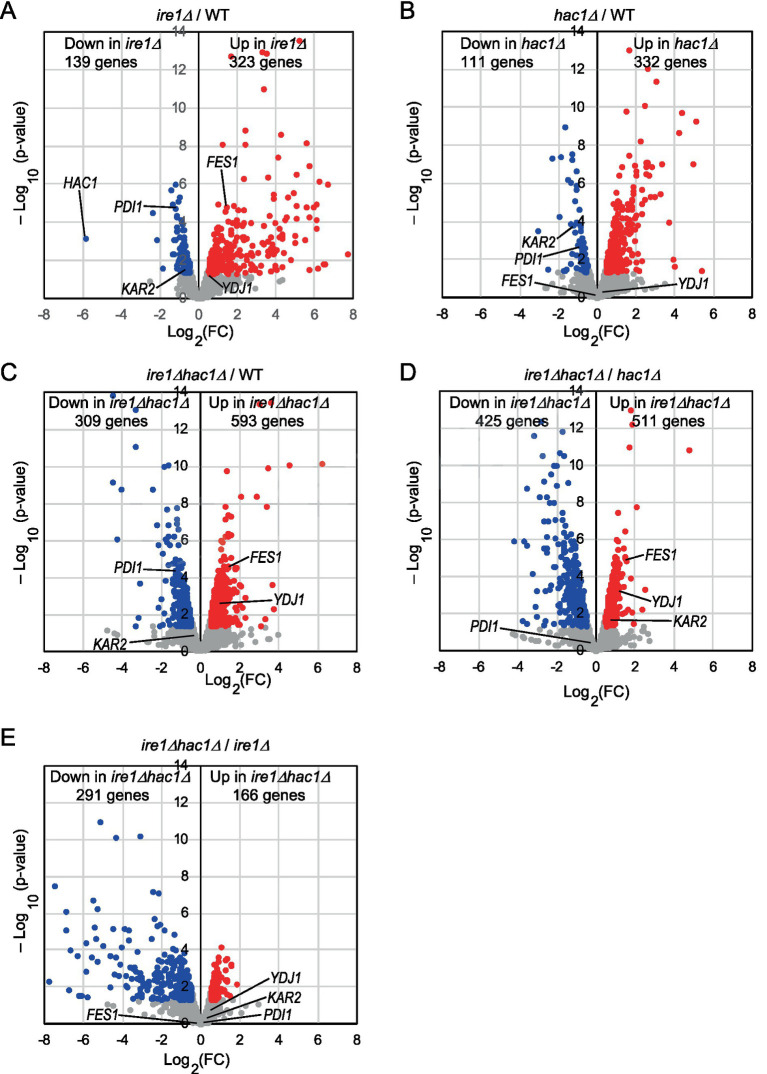
**(A–E)** Volcano plots displaying DEGs between two types of cells. WT, *ire1Δ* (*ire1Δ0*), *hac1Δ* (*hac1Δ0*), and *ire1Δhac1Δ* (*ire1Δ0 hac1Δ0*) versions of *P. pastoris* cells were cultured at 30°C under non-stress conditions, and their mRNA samples were subjected to RNA-seq analysis. See Supplementary Table S5 for the total data. In the volcano plots, the x-axis represents the Log_2_ of the fold change (FC), and the y-axis represents the negative decade logarithm of the value of *p*. DEGs (*p* < 0.05; Log_2_(FC) < −0.5 or > 0.5) are colored. We did not set the cut-off value for Log_2_(FC) greater than 0.5 because Ire1 was only moderately activated under our experimental conditions.

We cultured WT, *ire1Δ*, *hac1Δ*, and *ire1Δhac1Δ* cells under non-stress conditions for RNA extraction because, as aforementioned, the *IRE1*/*HAC1*-dependent UPR system is activated even without external stress stimuli in *P. pastoris* cells, albeit not strongly. We then subjected the cells to mRNA-seq analysis and presented the total data in Supplementary Table S5. In the volcano plots shown in Figures 2A–C, we compared the transcriptome of *ire1Δ* cells, *hac1Δ* cells, and *ire1Δhac1Δ* cells to that of WT cells and found that the expression of a number of genes is controlled by *IRE1* and *HAC1*. Consistent with our previous observations (Fauzee et al., 2020), the expression level of *HAC1* was positively and considerably regulated by *IRE1* (Figure 2A). Intriguingly, as shown in Figures 2D,E, the *ire1Δ* vs. *ire1Δhac1Δ* and *hac1Δ* vs. *ire1Δhac1Δ* comparisons also revealed many differentially expressed genes (DEGs). Thus, we deduced that at least partly, *IRE1* and *HAC1* act via different pathways in *P. pastoris*.

**Figure 3:**
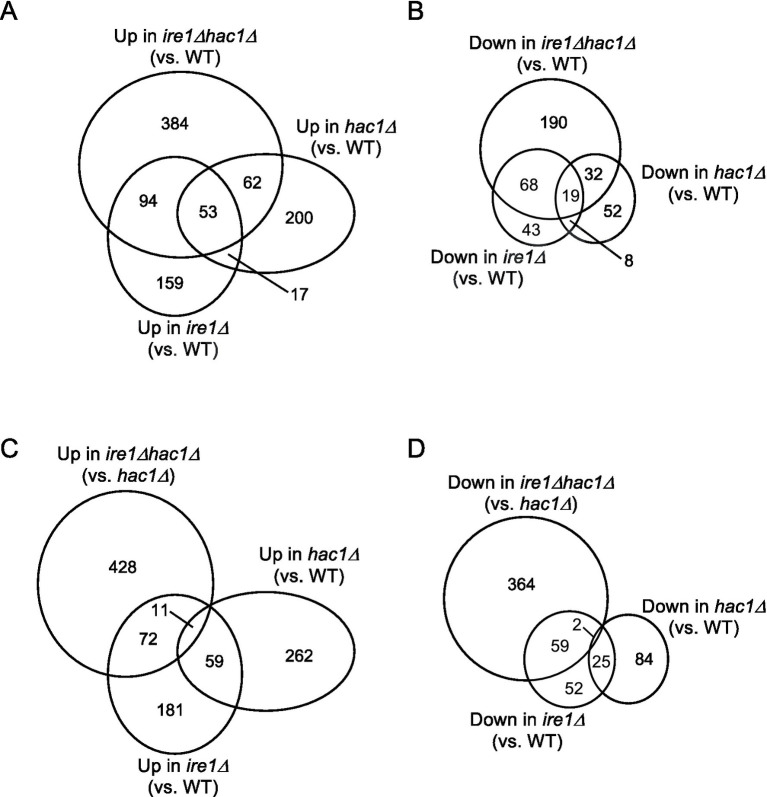
**(A–D)** Venn diagram presentation for DEGs between two types of cells. DEGs (*p* < 0.05; Log_2_(FC) < −0.5 or > 0.5) were extracted from the mRNA-seq data shown in Supplementary Table S5 and are presented as Venn diagrams.

This idea is supported by the Venn diagrams shown in Figure 3, which indicate that the DEGs of the *ire1Δ* mutation (*ire1Δ* vs. WT comparison) and those of the *hac1Δ* mutation (*hac1Δ* vs. WT comparison) overlapped only partially. The DEGs of the *ire1Δhac1Δ* mutation (*ire1Δhac1Δ* vs. WT comparison) also overlapped, but not perfectly, implying that *IRE1* and *HAC1* control the expression of various genes in both independent and interdependent manners (Figures 3A,B). To address the *HAC1*-independent function of *IRE1*, we also compared *ire1Δhac1Δ* vs. *hac1Δ* in Venn diagrams (Figures 3C,D). The DEGs in the *hac1Δ* vs. WT and *ire1Δhac1Δ* vs. *hac1Δ* comparisons overlapped only slightly (two induced and eleven repressed DEGs), supporting our proposal that the *HAC1*-dependent and the *HAC1*-independent functions of *IRE1* are distinct.

**Figure 4:**
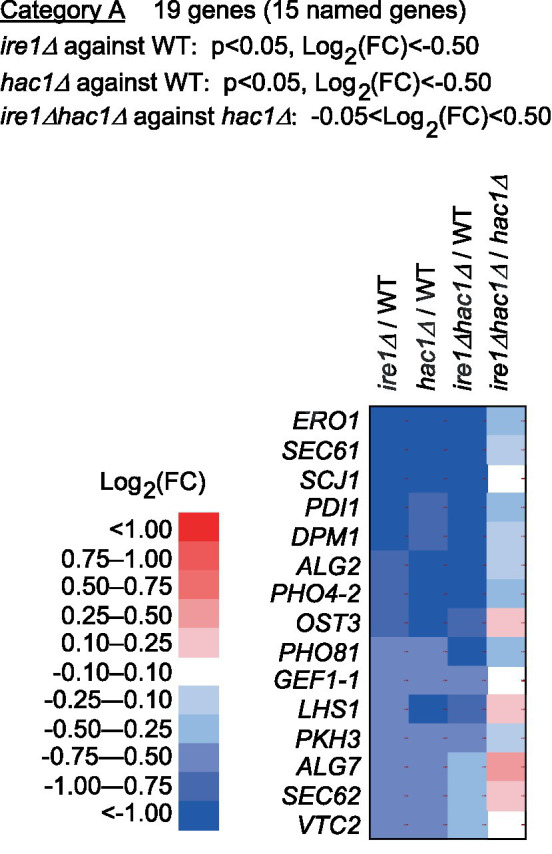
Genes cooperatively induced by *IRE1* and *HAC1*. The mRNA-seq data shown in Supplementary Table S5 were screened using the indicated criteria to extract the DEGs belonging to Category A. The heat map presents the expression profiles of the named genes in Category A, which are listed in Supplementary Table S6.

Next, we screened the total RNA-seq data for DEGs cooperatively induced by *IRE1* and *HAC1* (Category A) and controlled only by *IRE1* (Category B or C). Using the screening criteria presented in Figure 4, we selected 15 named genes as the Category-A DEGs (Figure 4 and Supplementary Table S6). Consistent with our expectation that Category-A DEGs are targets of the traditional UPR, many of them are known to be involved in ER protein translocation, folding, and modification (see the Discussion section for details).

**Figure 5:**
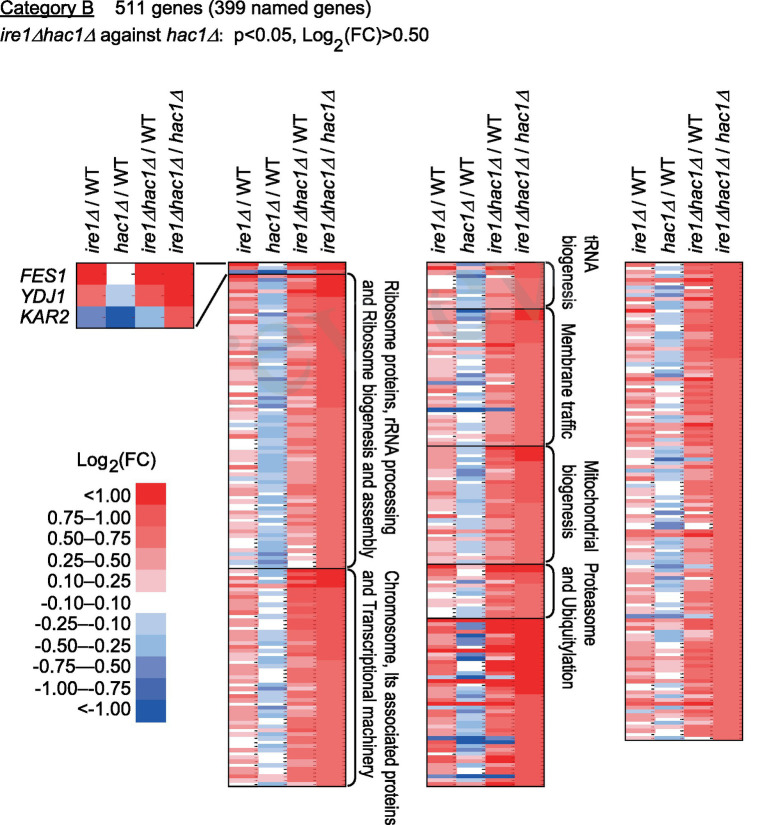
Genes suppressed by *IRE1* independently of *HAC1*. The mRNA-seq data shown in Supplementary Table S5 were screened using the indicated criteria to extract the DEGs belonging to Category B. The heat map presents the expression profiles of the named genes in Category B, which are listed in Supplementary Table S6.

Category B is a group of genes that were repressed by *IRE1* independent of *HAC1* (Figure 5 and Supplementary Table S6). The screening criteria for Category B are DEGs with elevated expression in comparison of *ire1Δhac1Δ* cells against *hac1Δ* cells. As shown in Figure 5, many of the Category-B genes were induced in *ire1Δ* cells and *ire1Δhac1Δ* cells compared to WT cells. As expected from our observations shown in Figure 1, *KAR2*, *YDJ1* and *FES1* fell into Category B. Expression of *FES1* and *YDJ1* was high in *ire1Δ* and *ire1Δhac1Δ* cells (Figure 5). Moreover, the expression of *KAR2* was considerably lowest in *hac1Δ* cells (Figure 5), presumably because it was induced by the HSR in *ire1Δ* and *ire1Δhac1Δ* cells.

According to the enrichment analysis shown in Supplementary Table S7, genes encoding ribosomal proteins and those related to ribosome biogenesis were highly enriched in Category B. The MA plot shown in Supplementary Figure S4 indicates that many of the ribosomal protein genes were abundantly expressed and induced in *ire1Δhac1Δ* cells compared to *hac1Δ* cells. It should also be noted that many genes related to the proteasome and ubiquitylation fell into Category B (Figure 5 and Supplementary Table S6).

**Figure 6:**
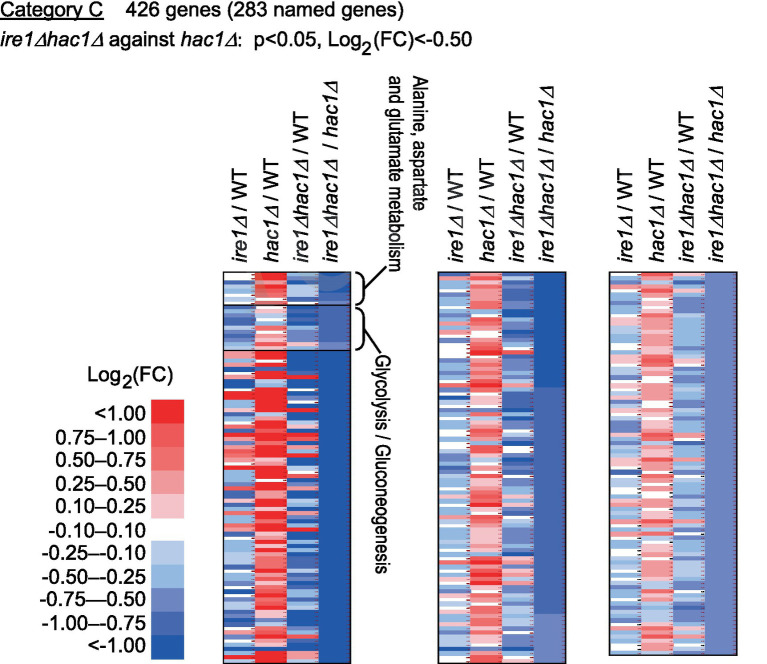
Genes induced by *IRE1* independently of *HAC1*. The mRNA-seq data shown in Supplementary Table S5 were screened using the indicated criteria to extract DEGs belonging to Category C. The heat map presents the expression profiles of the named genes in Category C, which are listed in Supplementary Table S6.

Category C is a group of genes induced by *IRE1* independent of *HAC1* (Figure 6 and Supplementary Table S6). As shown in Supplementary Table S7, genes for glycolysis/gluconeogenes and various metabolic pathways were highly enriched in Category C.

### Effect of the *ire1Δ* and *hac1Δ* mutations on cellular growth, protein aggregation, and stress tolerance in *Pichia pastoris* cells

**Figure 7:**
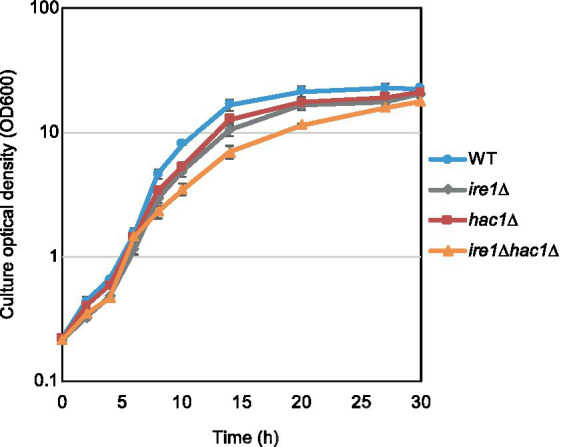
Growth profile of *P. pastoris* cells carrying the *ire1Δ* mutation and/or the *hac1Δ* mutation. After setting the initial OD_600_ values to approximately 0.3, the WT, *ire1Δ* (*ire1Δ0*), *hac1Δ* (*hac1Δ0*), and *ire1Δhac1Δ* (*ire1Δ0 hac1Δ0*) versions of *P. pastoris* cells were incubated at 30°C under non-stress conditions, and the optical density of the cultures was monitored.

In addition to the gene expression profile, we compared other phenotypes of the *ire1Δ* and *hac1Δ* mutations. In the experiment shown in Figure 7, we examined the growth of *P. pastoris* cells at 30°C in liquid media under non-stress conditions. Consistent with our previous study (Fauzee et al., 2020), *ire1Δ* cells grew slower than WT cells. We also noticed that the *hac1Δ* mutation retarded the growth of WT cells. Intriguingly, *ire1Δhac1Δ* cells grew slower than *ire1Δ* or *hac1Δ* cells. This observation supports our proposition that *IRE1* and *HAC1* act partly on different pathways.

**Figure 8:**
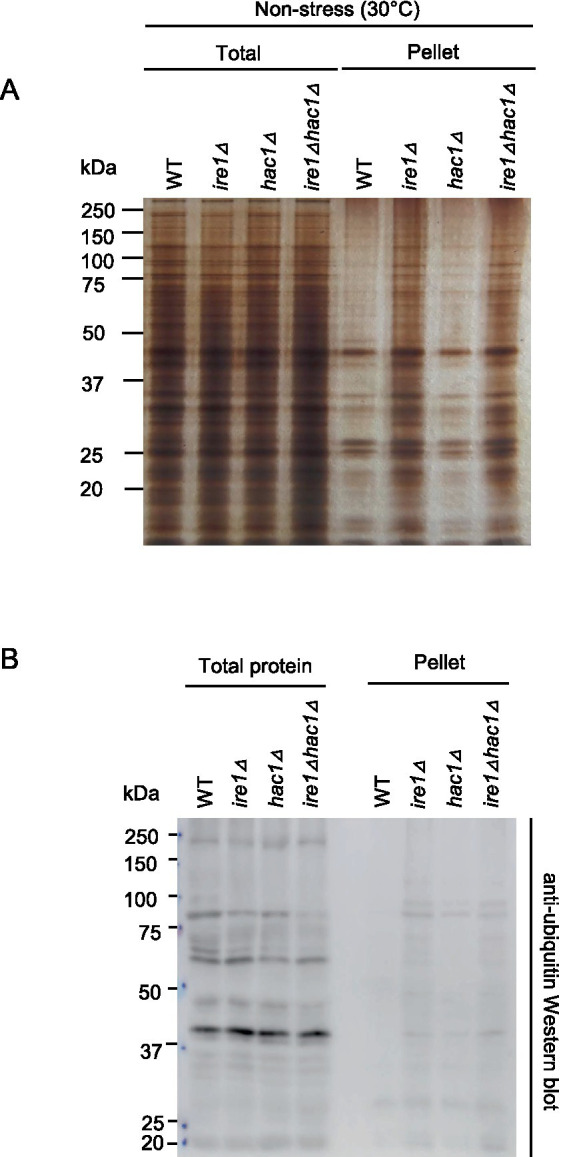
Induction of protein aggregation by the *ire1Δ* mutation. After culturing at 30°C under non-stress conditions, the WT, *ire1Δ* (*ire1Δ0*), *hac1Δ* (*hac1Δ0*), and *ire1Δhac1Δ* (*ire1Δ0 hac1Δ0*) versions of *P. pastoris* cells were harvested and lysed. The crude lysates (Total) were subjected to high-speed centrifugation, and pellet fractions (Pellet) were obtained. **(A)** Protein samples (Total: crude lysates corresponding to 6 μg protein; Pellet: preparation from crude lysates corresponding to 16 μg protein) were separated by SDS-PAGE and visualized by silver staining. **(B)** Protein samples (Total: crude lysates corresponding to 6 μg protein; Pellet: preparation from crude lysates corresponding to 16 μg protein) were subjected to SDS-PAGE, which was followed by anti-ubiquitin Western blotting.

As aforementioned, the *ire1Δ* mutation, but not the *hac1Δ* mutation, induced the HSR in *P. pastoris* cells. It is widely accepted that the HSR is a cellular protective response that is activated alongside the aggregation of proteins in the cytosol and/or nuclei (Chen and Qiu, 2020). Therefore, we monitored protein aggregation in cells carrying the *ire1Δ* and/or *hac1Δ* mutations. In the experiment shown in Figure 8, the cells were cultured at 30°C, and their lysates were fractionated by centrifugation. Figure 8A indicates that the pellet fractions of *ire1Δ* cells and *ire1Δhac1Δ* cells contained more abundant proteins than those of WT or *hac1Δ* cells. Anti-ubiquitin Western blot analysis showed that proteins in the pellet fractions were ubiquitylated, at least partly (Figure 8B). These observations strongly suggest a role of *IRE1* to prevent protein aggregation in the nuclei/cytosol.

**Figure 9:**
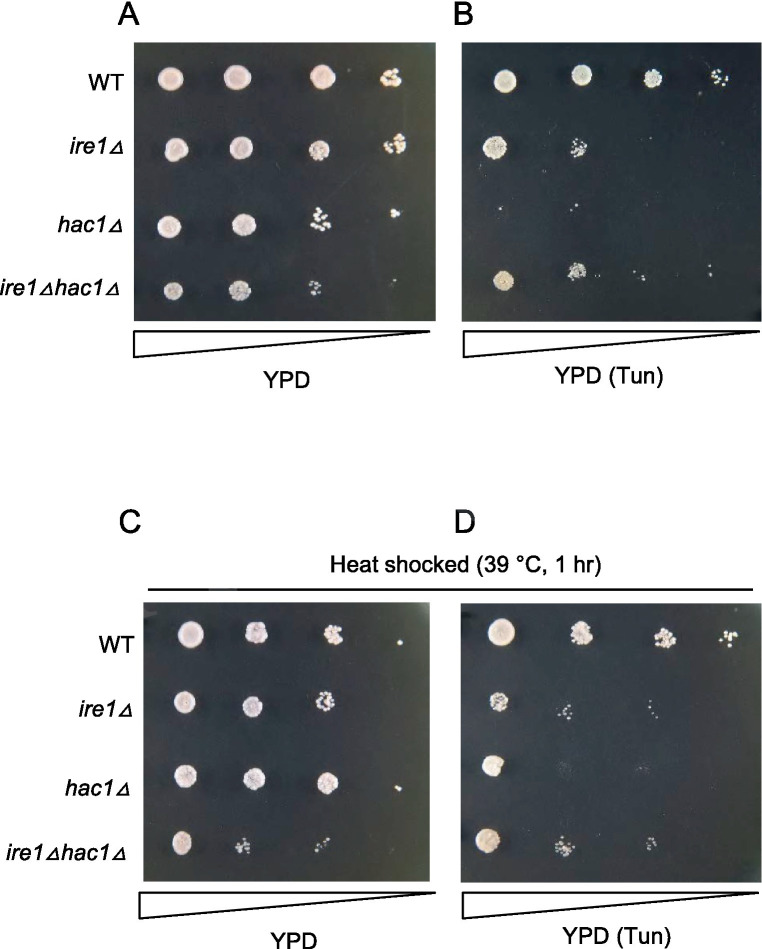
Tunicamycin sensitivity of *P. pastoris* cells carrying the *ire1Δ* mutation and/or the *hac1Δ* mutation. Cultures (OD_600_ = 1.0) of the WT, *ire1Δ* (*ire1Δ0*), *hac1Δ* (*hac1Δ0*), and *ire1Δhac1Δ* (*ire1Δ0 hac1Δ0*) versions of *P. pastoris* cells were 10-fold serially diluted and spotted onto YPD agar plates, which were incubated at 30°C for 2 days before being photographed. In panel **(A)**, cell were unstressed. In panels **(B,D)**, agar plates contained 4.0 μg/mL tunicamycin (Tun). In panels **(C,D)**, cultures were incubated at 39°C for 1 h before spotting.

Tunicamycin is an N-glycosylation-inhibiting antibiotic that is frequently used as a potent ER stressor. *S. cerevisiae* cells carrying the *ire1Δ* or *hac1Δ* mutation are known to be hypersensitive to tunicamycin. Figure 9 shows the growth of the *P. pastoris* cells on agar plates. In addition to the liquid medium (Figure 7), *ire1Δhac1Δ* cells appeared to grow slower than the other strains on agar plates under non-stress conditions (Figure 9A). As shown in Figure 9B, tunicamycin retarded the growth of cells carrying the *ire1Δ* and/or *hac1Δ* mutations more severely compared to that of WT cells. Moreover, *hac1Δ* cells were more susceptible to tunicamycin than *ire1Δ* and *ire1Δhac1Δ* cells (Figure 9B). In the experiment shown in Figures 9C,D, cells were incubated at 39°C for 1 h before being spotted onto agar plates. Intriguingly, this heat shock treatment partly mitigated the severe sensitivity of *hac1Δ* cells to tunicamycin (compare Figures 9D to B). As we mentioned above, the *ire1Δ* mutation induces the HSR in *P. pastoris* cells. Therefore, we presume that high tunicamycin sensitivity associated with UPR impairment, which is caused by the *ire1Δ* mutation or the *hac1Δ* mutation, is partially rescued by the HSR, which is induced by the *ire1Δ* mutation or heat shock treatment.

### Involvement of *IRE1* and *HAC1* in properties of heat-shocked *Pichia pastoris* cells

**Figure 10:**
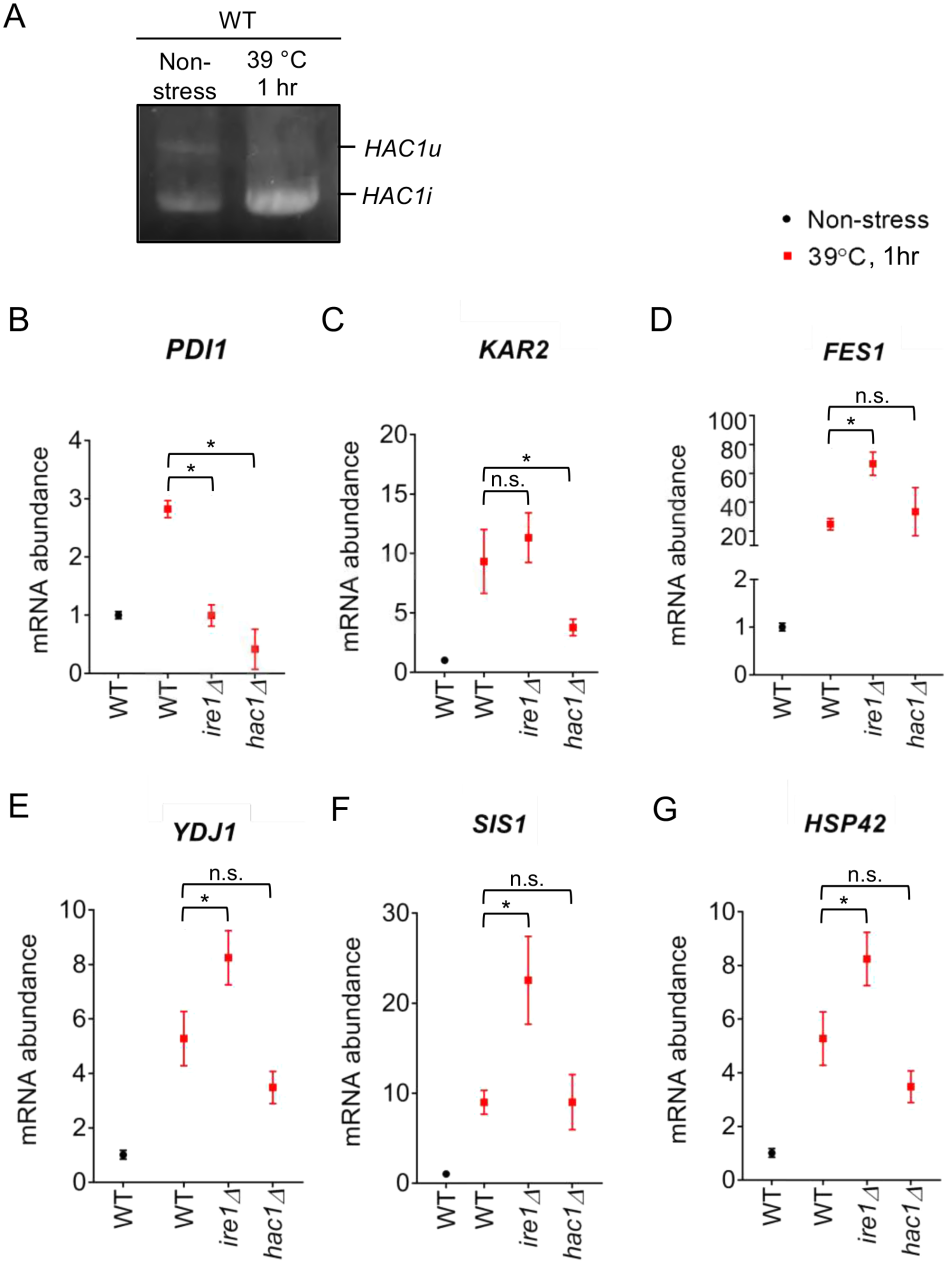
Heat shock-induced alteration of *HAC1* mRNA-splicing and gene-expression profiles of *P. pastoris* cells. **(A)** WT *P. pastoris* cells were cultured at 30°C under non-stress condition or shifted to 39°C for 1 h. RNA samples were subjected to RT-PCR to amplify the *HAC1* cDNA variants, which were then fractionated by agarose gel electrophoresis. **(B–G)** After culture at 30°C under non-stress conditions, WT, *ire1Δ* (*ire1Δ0*), *hac1Δ* (*hac1Δ0*), and *ire1Δhac1Δ* (*ire1Δ0 hac1Δ0*) versions of *P. pastoris* cells were shifted to 39°C for 1 h. Total RNA samples were subjected to RT-qPCR analysis using PCR primer sets that were specific to the indicated genes. Values are presented as relative to that of WT cells cultured at 30°C, which is set at 1.0. Dunnett’s test was performed using the data from WT cells as the control group. n.s.: not significant, *: significantly different (*p* < 0.05).

To elucidate the involvement of the UPR factors in the HSR more deeply, we examined the response of *P. pastoris* cells to high-temperature culturing. Figure 10A shows that splicing of *HAC1* mRNA was induced by a temperature shift from 30°C to 39°C, indicating UPR induction upon this temperature shift. Consistent with our proposal that *PDI* expression is positively regulated by the UPR but not by the HSR, it was induced by this temperature shift in WT cells but not in *ire1Δ* cells, *hac1Δ* cells, or *ire1Δhac1Δ* cells (Figure 10B).

Moreover, as shown in Figures 10D–G, this temperature shift also elevated the expression of the HSR marker genes *FES1*, *YDJ1*, *HSP42*, and *SIS1*. Because the temperature-dependent induction of these genes was stronger than that caused by the *ire1Δ* mutation at 30°C (compare Figures 10D,E to Figures 1E,F), we deduced that the *ire1Δ* mutation alone only moderately induces the HSR. Figures 10D–G also show that this temperature shift led to greater upregulation of the HSR marker genes in *ire1Δ* cells than in WT cells or *hac1Δ* cells. Thus, we presume that the HSR was additively or cooperatively induced by the temperature shift and the *ire1Δ* mutation. The expression pattern of *KAR2* (Figure 10C) can be explained by our proposition that in *P. pastoris* cells, *KAR2* is dually regulated by the UPR and HSR.

**Figure 11:**
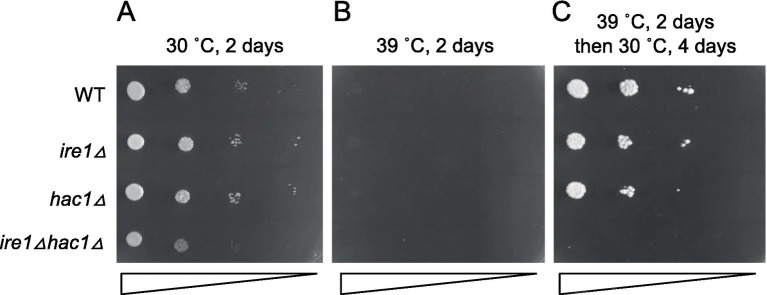
High-temperature sensitivity of *P. pastoris* cells carrying the *ire1Δ* and/or *hac1Δ* mutations. Cultures (OD_600_ = 1.0) of the WT, *ire1Δ* (*ire1Δ0*), *hac1Δ* (*hac1Δ0*), and *ire1Δhac1Δ* (*ire1Δ0 hac1Δ0*) versions of *P. pastoris* cells were 10-fold serially diluted and spotted onto YPD agar plates. **(A)** Agar plate was incubated at 30°C for 2 days and photographed. **(B)** Agar plate was incubated at 39°C for 2 days and photographed. **(C)** After incubation at 39°C for 2 days, the agar plate was incubated at 30°C for 4 days and photographed.

In the experiment shown in Figure 11, we examined the growth of cells on agar plates at different temperatures. All the strains were unable to grow at 39°C (Figure 11B). This agar plate was then shifted from 39°C to 30°C, resulting in the growth of all strains other than the *ire1Δhac1Δ* strain (Figure 11C). Therefore, we assume that *IRE1* and *HAC1* confer heat resistance to *P. pastoris* cells in different ways.

**Figure 12:**
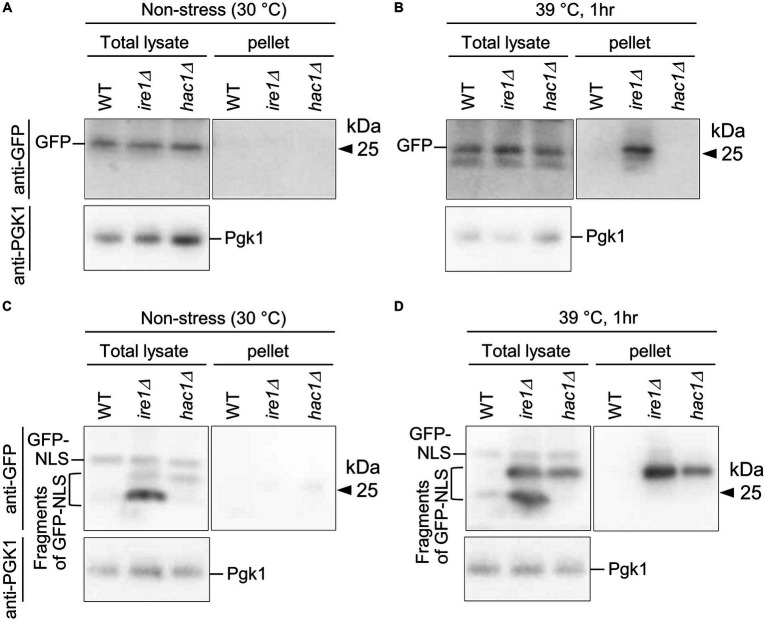
Aggregation and truncation of GFP and GFP-NLS in *P. pastoris* cells carrying the *ire1Δ* mutation or *hac1Δ* mutation. After culturing at 30°C under non-stress conditions **(A,C)** or shifting to 39°C for 1 h **(B,D)**, the WT, *ire1* (*ire1Δ0*), and *hac1Δ* (*hac1Δ0*) versions of *P. pastoris* cells expressing GFP **(A,B)** or GFP-NLS **(C,D)** were harvested and lysed. The crude lysates (Total) were subjected to high-speed centrifugation, and pellet fractions (Pellet) were obtained. Protein samples (Total: crude lysates corresponding to 6 μg protein; Pellet: preparation from crude lysates corresponding to 16 μg protein) were subjected to SDS-PAGE, which was followed by anti-GFP Western blotting. Two anti-GFP blot panels in each of **(A–D)** are from the same membrane image. Anti-Pgk1 Western blots are used as loading control.

In the final part of our study, we checked cytosolic/nuclear protein aggregation using GFP as a marker protein. First, WT, *ire1Δ*, and *hac1Δ* cells expressing GFP were cultured at 30°C or shifted to 39°C before cell lysis, the products of which were then high-speed centrifuged (Figures 12A,B). Figure 12B shows that the high-temperature treatment at 39°C caused GFP aggregation in *ire1Δ* cells. This observation supports our proposition that the *ire1Δ* mutation aggravates protein aggregation in the cytosol and/or nuclei of cells. Next, a similar experiment was performed using cells expressing GFP that carries an NLS at the C-terminus (GFP-NLS). Figure 12C shows that, in addition to full-length GFP-NLS, shorter versions (probably degraded fragments) of GFP-NLS were detected abundantly from *ire1Δ* cells, and to a lesser extent, from *hac1Δ* cells. A GFP-NLS fragment was delivered to the pellet fractions at least partly when cells were sifted to 39°C before lysis. Therefore, we deduce that there exits an Ire1-dependent system to cope with aberrant proteins accumulated in the cytosol and/or nuclei.

## Discussion

As described thus far, it is widely believed that in *S. cerevisiae*, *HAC1* mRNA is the sole target of Ire1. Tam et al. (2014) proposed that the RIDD occurs in *S. cerevisiae* cells, but this observation was not reproduced in our study (data not shown). Moreover, *HAC1* mRNA is virtually functionless unless it is spliced by Ire1. Thus, the functions of *IRE1* and *HAC1* are severely interdependent. However, here we note that this insight is not applicable to *P. pastoris*. Our observations cumulatively indicate that in *P. pastoris* cells, *IRE1* and *HAC1* play both interdependent and independent roles.

Category A is a group of genes induced by the traditional UPR, for which *IRE1* and *HAC1* function cooperatively (Figure 4). Genes in Category A included those encoding the ER-located molecular chaperone (*LHS1*), factors for disulfide bond formation in the ER (*ERO1* and *PDI1*), factors for glycosylation (*DPM1*, *OST3*, *ALG2*, and *ALG7*), and factors for protein translocation into the ER (*SEC61* and *SEC62*). We deduce that Category-A genes are transcriptionally induced by the translation product of spliced *HAC1* mRNA, which acts as a nuclear transcription factor. Using a DNA microarray technique, Graf et al. (2008) listed genes that were induced by DTT treatment and artificial expression of spliced *HAC1* mRNA in *P. pastoris* cells. As expected, Category-A genes were included in Grafs’ list. The number of genes in Category A in our study was smaller than that in Grafs’ list, probably because, in our case, *P. pastoris* cells were cultured under non-stress conditions and provoked the UPR only moderately.

Moreover, the list of Category-A genes overlaps with that of the UPR target genes in *S. cerevisiae* cells (Travers et al., 2000; Kimata et al., 2006). In this context, the UPR in *P. pastoris* cells and *S. cerevisiae* cells has the same biological meaning of enhancing the activity of the ER and protein secretory pathway. Nevertheless, it should be also noted that the UPR target genes in these two species were not identical. For instance, membrane lipid biosynthesis genes, such as *INO1* and *SCS3*, were not induced by the UPR in *P. pastoris* cells (Supplementary Table S5). Unlike the case of *S. cerevisiae* cells (Schuck et al., 2009; Nguyen et al., 2022), expansion of the ER membrane may not be an outcome of the UPR in *P. pastoris* cells.

Meanwhile, the main argument of the present study is that in *P. pastoris* cells, Ire1 also functions independently of *HAC1*. Here we note that the *ire1Δ* mutation, but not the *hac1Δ* mutation, provoked the HSR and protein aggregation. Because aggregated proteins were at least partially ubiquitylated, we assume that they were formed in the cytosol and/or nuclei. Therefore, it is likely that in *P. pastoris* cells carrying the *ire1Δ* mutation, cytosolic and/or nuclear protein aggregation provokes the HSR. To the best of our knowledge, the role of Ire1 to suppress cytosolic and/or nuclear protein aggregation and the HSR has not been previously reported in any eukaryotic species. As shown in Figure 7, the *ire1Δ* mutation retarded cellular growth, even in the *hac1Δ* background. This observation supports the physiological importance of the *HAC1*-indepencent function of Ire1 in *P. pastoris*.

Category B is a group of genes induced by the *ire1Δ* mutation in the *hac1Δ* background (Figure 5). Probably because the *ire1Δ* mutation only modestly provoked the HSR, certain genes that are deduced to be heat shock genes, namely, genes for cytosolic molecular chaperones and their co-factors, did not fall into Category B. Nevertheless, Category B is composed of a number of other genes, including those encoding ribosomal proteins and ribosome biogenesis factors. According to Tye et al. (2019), aberrant ribosome biogenesis yields unassembled ribosomal proteins, which are proteotoxic and induce the HSR. Therefore, Ire1 may suppress the expression of ribosomal proteins and ribosome biogenesis factors, thus leading to the attenuation of the HSR. As shown in Supplementary Figure S4, ribosomal proteins were abundantly expressed in cells. Thus, we assume that the suppression of ribosomal protein expression by Ire1 is important for reducing the protein load into the cytosol and/or the nuclei, which may result in attenuation of the HSR.

Some genes related to the proteasome and ubiquitylation were also grouped into Category B. Upregulation of the ubiquitin/proteasome-dependent protein-degradation pathway may be a cellular response to cope with cytosolic and/or nuclear protein aggregation.

Nevertheless, the proximal role of Ire1 besides splicing *HAC1* mRNA in *P. pastoris* cells has not been clarified. One possibility is the RIDD, through which Ire1 decreases the cellular abundance of specific mRNAs, many of which encode ER client proteins, independent of *HAC1* (Coelho and Domingos, 2014). Indeed, as aforementioned, the *ire1Δ* mutation increased the expression of a number of genes, namely, Category B genes, in the *hac1Δ* background. However, it should also be noted that genes for ER client proteins were not enriched in Category B. Moreover, according to the structure prediction by Li et al. (Li et al., 2021), the RNase domain of Ire1 in *P. pastoris*, as well as that in *S. cerevisiae*, has a narrow substrate specificity and is unlikely to perform the RIDD. As proposed in a study on mammalian cells (Urano et al., 2000), it may also be possible that the kinase domain of Ire1 performs not only auto-phosphorylation but also phosphorylation of other proteins.

What is the physiological meaning of Ire1, which is widely believed to be a factor to cope with ER stress, to mitigate cytosolic and/or nuclear protein aggregation and the HSR? According to Hamdan et al. (2017), ER stress totally damages the cellular protein-folding status, thus leading to protein aggregation not only in the ER but also in the cytosol in *S. cerevisiae*. On the other hand, in some human neurodegenerative diseases including Parkinson’s disease, proteins aggregated in the cytosol are thought to trigger ER stress (Melo et al., 2018). In other words, cytosolic protein aggregation can be a cause and an outcome of ER stress.

In this study, we also demonstrated another intriguing relationship between the UPR and HSR. Based on our observation shown in Figure 9, we propose that the high sensitivity of UPR-deficient cells to ER stress can be partly rescued by inducing the HSR in both *P. pastoris* and *S. cerevisiae* cells (Liu and Chang, 2008). However, whether this observation can be explained solely by the expression level of *KAR2*, which is positively regulated by both the UPR and HSR, has not been clarified.

Meanwhile, according to our observations presented here, not only Ire1 but also *HAC1* plays a role(s) other than performing the traditional UPR, in which Ire1 splices *HAC1* mRNA, in *P. pastoris* cells. Since *HAC1* worked both dependently and independently of Ire1, we assume not only the spliced form but also the unspliced form of *HAC1* mRNA has a biological function(s), which should be addressed in future studies.

Because, in general, high temperature impairs protein folding, heat stress likely induces both the HSR and UPR. Nevertheless, the UPR is only slightly induced at high temperatures in *S. cerevisiae* cells (Hata et al., 2022). On the other hand, here we observed strong activation of Ire1 in heat-stressed *P. pastoris* cells (Figure 10). The expression of the UPR marker gene *PDI1* was elevated by a temperature shift from 30°C to 39°C dependently on both *IRE1* and *HAC1*. Moreover, this temperature shift strongly induced the HSR, which was mitigated by Ire1. We also demonstrated the involvement of Ire1 in the heat resistance of *P. pastoris* cells (Figure 11).

Also in plant cells, heat stress considerably activates Ire1, which then splices and matures bZIP60 mRNA (Deng et al., 2011; Li et al., 2012). Moreover, bZIP60 induces the transcription factor HSFTF13, which upregulates the HSR (Li et al., 2020). Therefore, unlike the case of *P. pastoris*, Ire1 contributes to inducing the HSR under heat stress conditions in plant cells.

In conclusion, we demonstrated a new function of Ire1 through genetic analyses of *P. pastoris* cells. In addition to splicing of *HAC1* mRNA, Ire1 suppressed cytosolic and/or nuclear protein aggregation and the HSR, possibly by avoiding excessive production of ribosomal proteins. The role of Ire1 to mitigate the HSR was also observed under heat stress conditions, and Ire1 conferred heat resistance to *P. pastoris* cells. Further studies are required to elucidate whether this insight is applicable to other yeast and fungal species.

### Importance of this research

In eukaryotic cells, secretory and cell-surface proteins are mainly folded in the endoplasmic reticulum (ER) while cytosolic and nuclear proteins are folded in the cytosol. Dysfunction of the ER, namely, ER stress, is accompanied by the accumulation of unfolded proteins in the ER and provokes the unfolded protein response (UPR), by which ER-located molecular chaperones and their co-factors are transcriptionally induced. The ER-located transmembrane protein Ire1 is the most prominent ER stress sensor that triggers the UPR. On the other hand, the heat shock response (HSR) is provoked by the accumulation of unfolded proteins in the cytosol or the nuclei, resulting in transcriptional induction of cytosolic molecular chaperones and their co-factors. Here we show that in addition to UPR induction, Ire1 functions to attenuate cytosolic/nuclear protein aggregation and the HSR in cells of the methylotrophic yeast *Pichia pastoris* (syn. *Komagataella phaffi*). Moreover, Ire1 was activated by heat stress to confer heat resistance to *P. pastoris* cells. Our findings presented here reveal a previously unknown case in which the UPR and HSR are tightly coupled.

## Data availability statement

The datasets presented in this study can be found in online repositories. The names of the repository/repositories and accession number(s) can be found in the article/Supplementary material.

## Author contributions

YK led the project and wrote the final manuscript. YF designed and performed the experiments. YF and YY constructed yeast strains. All authors have read and approved the final manuscript.

## Funding

This research was funded by JSPS KAKENHI (grant number 22 K19135) to YK and a research grant to YK from Noda Institute for Scientific Research.

## Conflict of interest

The authors declare that the research was conducted in the absence of any commercial or financial relationships that could be construed as a potential conflict of interest.

## Publisher’s note

All claims expressed in this article are solely those of the authors and do not necessarily represent those of their affiliated organizations, or those of the publisher, the editors and the reviewers. Any product that may be evaluated in this article, or claim that may be made by its manufacturer, is not guaranteed or endorsed by the publisher.
