# Supplementary material for: Endoplasmic stress sensor Ire1 is involved in cytosolic/nuclear protein quality control in Pichia pastoris cells independent of HAC1
Source: Front Microbiol. 2023 Jun 20;14:1157146. doi: 10.3389/fmicb.2023.1157146 (PMC10321714; doi:10.3389/fmicb.2023.1157146)
Supplement: Supplementary file 3 [file Data_Sheet_1.pdf]

Table S1

Name	Sequence	Description
gRNA-IRE1 DNA	tcaattgaacaactatcaaaacaccatgGCAGATCTGATGAGTCCGTGAGGACGAAACGAGTAAGCTCGTCATCTGCGTTTACTCATAATGGTTTTAGAGCTAGAAATAGCAAGTTAAAATAAGGCTAGTCCGTTATCAACTTGAAAAAGTGGCACCGAGTCGGTGCTTTTGGCCGGCATGGTCCCAGCCTCCTCGCTGGCGCCGGCTGGGCAACATGCTTCGGCATGGCGAATGGGACAGCTTTGGACTgcttttagtgtacatctgataatatagt	Synthetic DNA fragment carrying the guide RNA targeting *IRE1*
gRNA-HAC1 DNA	tcaattgaacaactatcaaaacaccatgGTAATGCTGATGAGTCCGTGAGGACGAAACGAGTAAGCTCGTCCATTACAGCAGGCTCCATCGGTTTTAGAGCTAGAAATAGCAAGTTAAAATAAGGCTAGTCCGTTATCAACTTGAAAAAGTGGCACCGAGTCGGTGCTTTTGGCCGGCATGGTCCCAGCCTCCTCGCTGGCGCCGGCTGGGCAACATGCTTCGGCATGGCGAATGGGACAGCTTTGGACTgcttttagtgtacatctgataatatagt	Synthetic DNA fragment carrying the guide RNA targeting *HAC1*

Table S1: Synthetic DNA fragments used to generate the guide RNA/Cas9 expression plasmids

Target gene-specific sequences are indicated by the red letters. Sequences for hybridization to BbsI-digested BB3cK_pGAP_23*_pPFK300_Cas9 for the Gibson assembly ligation are indicated by lowercase letters.

Table S2

Name	Direction	Sequence	Target	Final product
I1	Forward	**GCTTAATGGAAATCATTTGGTTCT**	5’-flanking region of *IRE1*	Donor DNA for the *ire1Δ0* allele
I4	Reverse	**tgaatggctacGATATAATTATCACTCACTGCAGG** (small letters: Sequence for annealing to the I3/I2 PCR product)	5’-flanking region of *IRE1*	
I3	Forward	**ataattatatcGTAGCCATTCAACTATGCACATAC** (small letters: Sequence for annealing to the I1/I4 PCR product)	3’-flanking region of *IRE1*	
I2	Reverse	**CTCCTTGATACTTCTATTATACTT**	3’-flanking region of *IRE1*	
I5	Forward	**CCTAGCCCTTTGAGTGCGTCTAGA**	5’-flanking region of *IRE1*	
I6	Reverse	**GAATATTCTTTTTCCTTTCTTCTC**	3’-flanking region of *IRE1*	
H1	Forward	**GATGGGAGCACATCAAGTGTAC**	5’-flanking region of *HAC1*	Donor DNA for the *hac1Δ0* allele
H6	Reverse	**ttaaatcaaaTTTTCTGCGATCTGATTCGACTAAG** (small letters: Sequence for annealing to the H5/H2 PCR product)	5’-flanking region of *HAC1*	
H5	Forward	**gatcgcagaaaATTTGATTTAAATGACTTTGTATT** (small letters: Sequence for annealing to the H1/H6 PCR product)	3’-flanking region of *HAC1*	
H2	Reverse	**CCCAAATTTCAATGCTTCCC**	3’-flanking region of *HAC1*	
H7	Forward	**CGCGCTATTCACCGCGAATAC**	5’-flanking region of *HAC1*	
H8	Reverse	**CTAACCTGTAAAGAGCTTGGC**	3’-flanking region of *HAC1*	

Table S2: Oligonucleotide primers used for PCR to generate donor DNAs for the CRISR/Cas9-based genome editing

Table S3

Name	Direction	Sequence	Target	Product
H9	Forward	**AGACAGCTAGCCCACTTCCACCTCG**	*HAC1*	*HAC1* fragment (first half)
H10	Reverse	**gggcctccatgtcCTCCTGCTTGATAGATGTGCTC** (small letters: Sequence for annealing to the H13/H14 PCR product)	*HAC1*	
H13	Forward	**atcaagcaggagGACATGGAGGCCCAGAATACCC** (small letters: Sequence for annealing to the H9/H10 PCR product)	*kanMX*	*kanMX*
H14	Reverse	**atggagctgtagAGTATAGCGACCAGCATTCAC** (small letters: Sequence for annealing to the H11/H12 PCR product)	*kanMX*	
H11	Forward	**gtcgctatactCTACAGCTCCATCAGGTTCCATCA** (small letters: Sequence for annealing to the H13/H14 PCR product)	*HAC1*	*HAC1* fragment (latter half)
H12	Reverse	**GCATTAGCGGTAAATGGTGCTGC**	*HAC1*	
H3	Forward	**GAGCAAAGACGGAAGAAGAAAAGG**	*HAC1*	*hac1Δ::kanMX* module
H4	Reverse	**TTAACTACGCGTCTCGAACAAGGG**	*HAC1*	

Table S3: Oligonucleotide primers used for PCR to generate the *hac1Δ::kanMX* gene-deletion module

Table S4

Name	Purpose	Direction	Target	Sequence
P1	RT		*HAC1* (2nd exon)	**CATTAGCGGTAAATGGTGCTG**
P2	RT		Universal	**TTTTTTTTTTTTTTTTTT**
P3	PCR	Forward	*HAC1* (1st exon)	**AGACAGCTAGCCCACTTCCACCTCG**
P4	PCR	Reverse	*HAC1* (2nd exon)	**CATTAGCGGTAAATGGTGCTG**
P7	qPCR	Forward	*KAR2*	**TGCTTGGTAAATTCGAGCTG**
P8	qPCR	Reverse	*KAR2*	**CAACTTGAGGAGTACCTCTTGGA**
P9	qPCR	Forward	*PDI1*	**GGAAAGGCCCACGATGAAGTTGTC**
P10	qPCR	Reverse	*PDI1*	**GCATCCTCATCATTGGCGTAAAGAGTAG**
P11	qPCR	Forward	*FES1*	**CTCAGGATGAGGAATCCAAGA**
P12	qPCR	Reverse	*FES1*	**GGGCCTCCAAACAACTGAG**
P13	qPCR	Forward	*YDJ1*	**GACAAATTGGCCCCATGAT**
P14	qPCR	Reverse	*YDJ1*	**CTTCTCCGTTACAAACATCACATC**

Table S4: Oligonucleotide primers used for RT-PCR and RT-qPCR analyses

Table S7

Category B

Pathway name	p-value
Ribosome	9.278e-9
Ribosome biogenesis in eukaryotes	2.837e-8
Nucleotide excision repair	0.000007329
Basal transcription factors	0.00003546
Protein processing in endoplasmic reticulum	0.00006509
Proteasome	0.00006038
DNA replication	0.0003414
Autophagy	0.0002949
mRNA surveillance pathway	0.0002897
Folate biosynthesis	0.0008520

Category C

Pathway name	p-value
Glycolysis / Gluconeogenesis	2.016e-10
Alanine, aspartate and glutamate metabolism	8.425e-9
Starch and sucrose metabolism	5.959e-8
Nitrogen metabolism	5.677e-7
Fructose and mannose metabolism	3.384e-7
Methane metabolism	0.000001053
Steroid biosynthesis	0.000002897
Sphingolipid metabolism	0.00003465
MAPK signaling pathway	0.000004962
Arginine biosynthesis	0.00007968

Table S7: KEGG pathway enrichment of the Category-B and Category-C genes

Lists of named genes (Table S6) were input to the YeastEnrichr WEB site (https://maayanlab.cloud/YeastEnrichr/), and the results from the data analysis (dataset: *S. cerevisiae* KEGG 2019 pathways [Wixon and Kell (2000) *Yeast* Vol.17, 48-55]) are indicated.

Figure S1

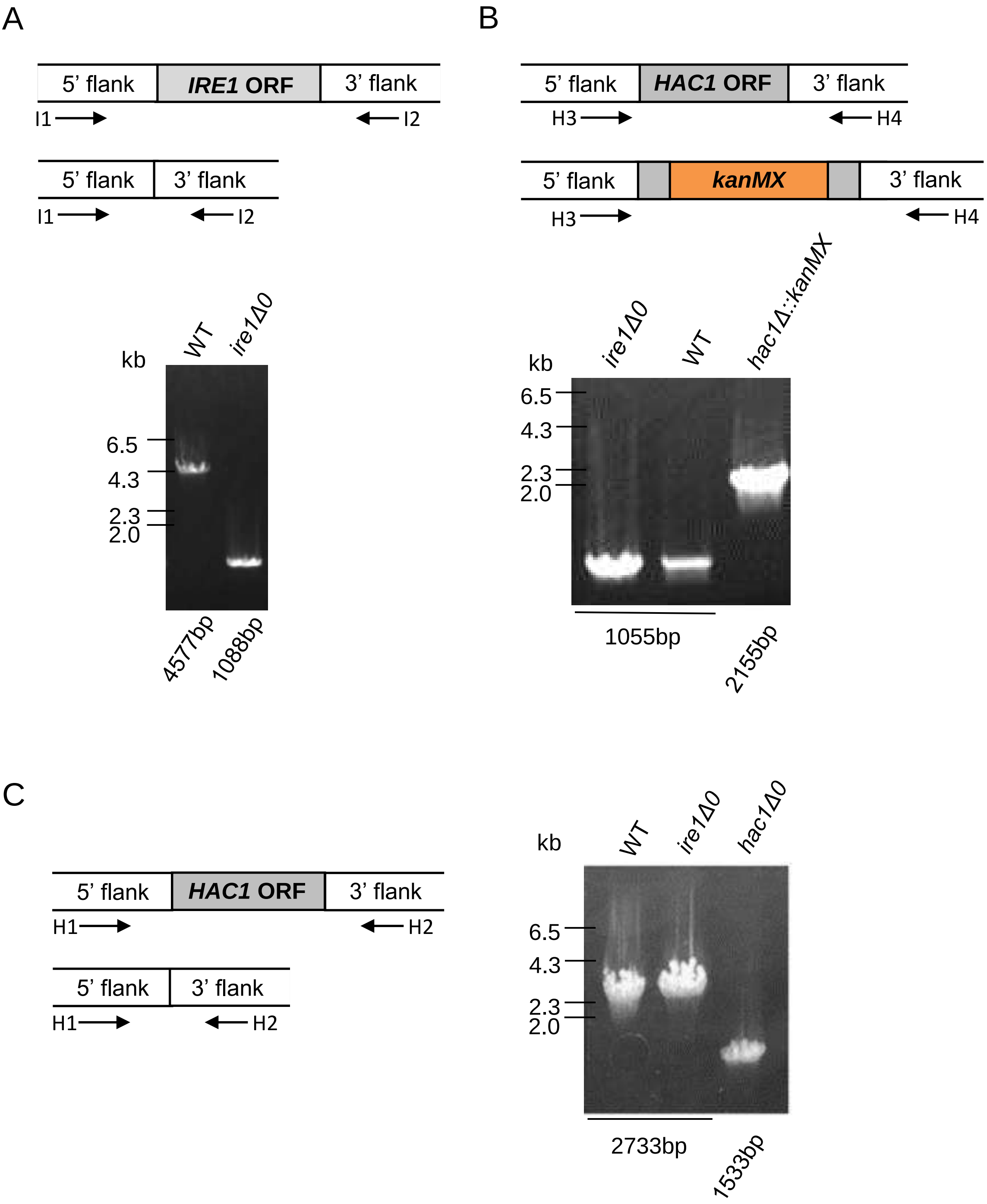
A
5' flank
IRE1 ORF
3' flank
I1
I2
5' flank
3' flank
I1
I2
kb
WT
ire1Δ0
6.5
4.3
2.3
2.0
4577bp
1088bp
B
5' flank
HAC1 ORF
3' flank
H3
H4
5' flank
kanMX
3' flank
H3
H4
kb
ire1Δ0
WT
hac1Δ::kanMX
6.5
4.3
2.3
2.0
1055bp
2155bp
C
5' flank
HAC1 ORF
3' flank
H1
H2
5' flank
3' flank
H1
H2
kb
WT
ire1Δ0
hac1Δ0
6.5
4.3
2.3
2.0
2733bp
1533bp

Figure S1: Construction of *P. pastoris ire1Δ* and *hac1Δ* mutants

(A) The *ire1Δ0* mutation is a full-length deletion of the coding sequence of the *IRE1* gene. To confirm that the *ire1Δ0* mutation was introduced as expected, genomic DNA samples were used as templates for PCR with the oligonucleotide primer set I1 and I2 (Table S2), and the PCR products were run on agarose gel. (B) To construct the *hac1::kanMX* mutant, the *kanMX* marker was inserted into the *HAC1* gene. To confirm that the *hac1::kanMX* mutation was introduced as expected, genomic DNA samples were used as templates for PCR with the oligonucleotide primer set H3 and H4 (Table S3), and the PCR products were run on agarose gel. C. The *hac1Δ0* mutation is a full-length deletion of the coding sequence of the *HAC1* gene. To confirm that the *hac1Δ0* mutation was introduced as expected, genomic DNA samples were used as templates for PCR with the oligonucleotide primer set H1 and H2 (Table S2), and the PCR products were run on agarose gel.

Figure S2

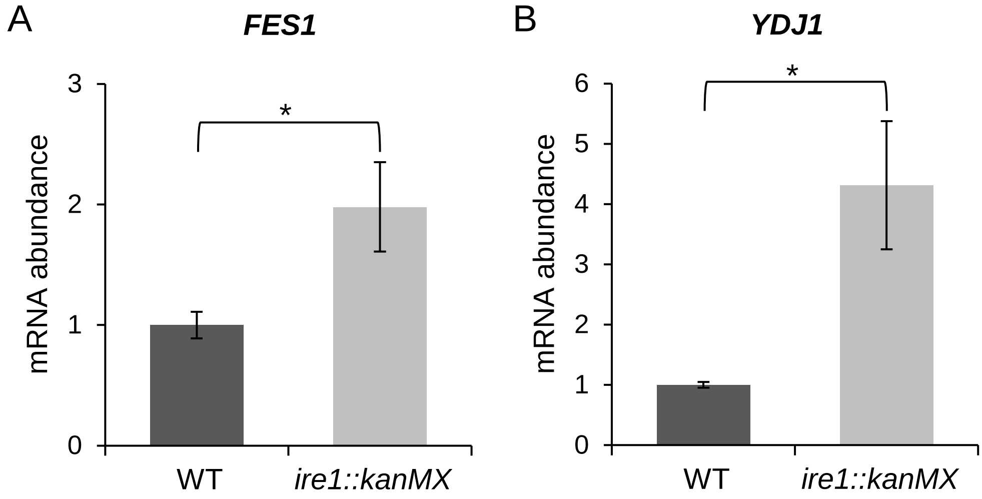

Figure S2: Induction of HSR reporter genes by the *ire1::kanMX* mutation

WT and *ire1Δ* (*ire1::kanMX*) versions of *P. pastoris* cells were cultured under non-stress conditions, and their RNA samples were subjected to RT-qPCR analysis using PCR primer sets that were specific to the indicated genes. Values are presented as relative to that of WT cells, which is set at 1.0. *: $p<0.05$.

Figure S3

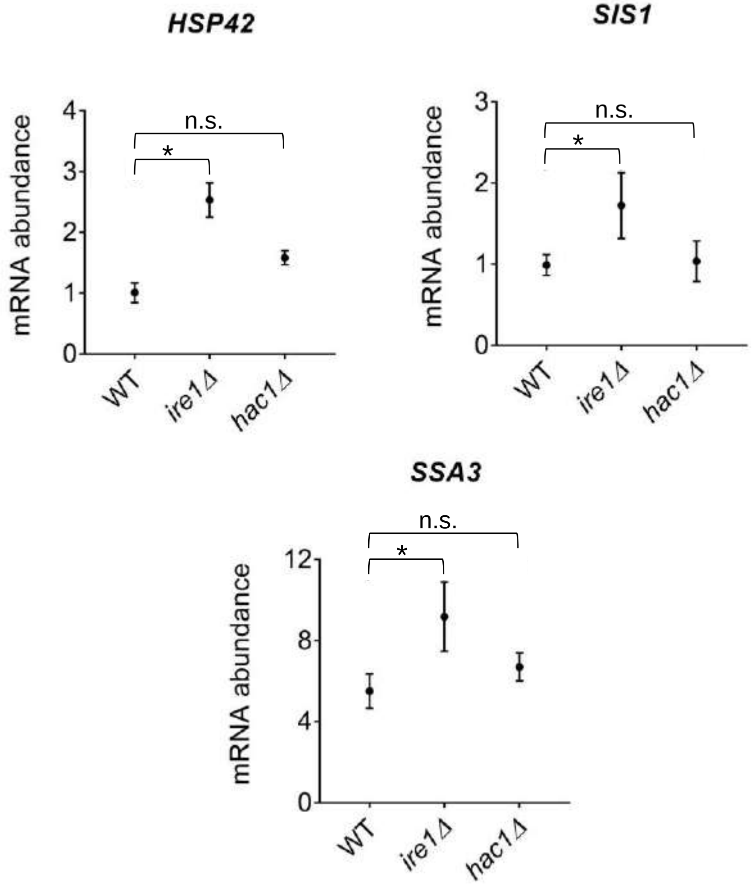

Figure S3: Expression profiles of other HSR-marker genes in *P. pastoris* cells carrying the *ire1Δ* mutation or *hac1Δ* mutation

WT, *ire1Δ* (*ire1Δ0*), and *hac1Δ* (*hac1::kanMX*) versions of *P. pastoris* cells were cultured at 30 ° C under non-stress conditions, and total RNA samples were subjected to RT-qPCR analysis using PCR primer sets that were specific to the indicated genes. Values are presented as relative to that of non-stressed WT cells, which is set at 1.0. Dunnett's test was performed using the data from WT cells as the control group. n.s.: not significant, *: significantly different ($p<0.05$).

Figure S4

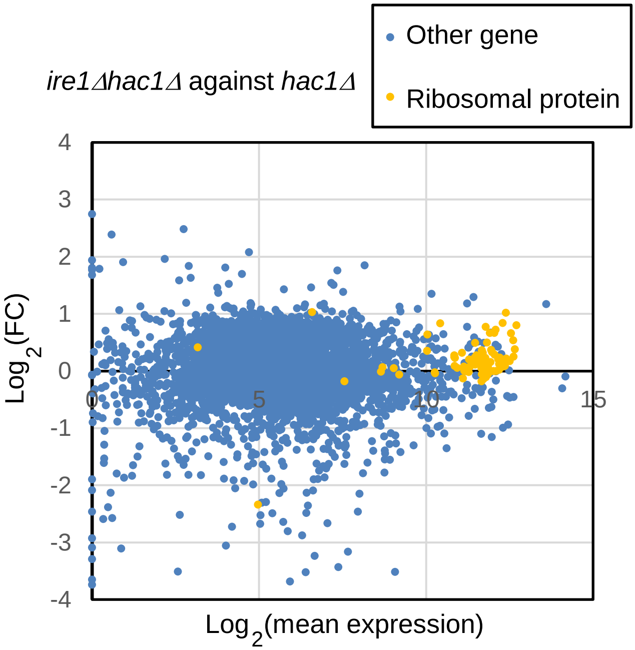

Figure S4: MA plot presentation for DEGs between *ire1Δhac1Δ* cells and *hac1Δ* cells

The mRNA-seq data shown in Table S1 are expressed as an MA plot, in which the x-axis represents Log_2 of the mean expression level (TPM) and the y-axis represents Log_2 of the FC.
